# Increased Fas and Bcl-2 Expression on Peripheral Blood T and B Lymphocytes from Juvenile-Onset Systemic Lupus Erythematosus, but not from Juvenile Rheumatoid Arthritis and Juvenile Dermatomyositis

**DOI:** 10.1080/17402520600877786

**Published:** 2006

**Authors:** Bernadete L. Liphaus, Maria H. B. Kiss, Solange Carrasco, Claudia Goldenstein-Schainberg

**Affiliations:** 1 Rheumatology Unit Department of Pediatrics School of Medicine Children's Institute, University of São Paulo Brazil; 2 Rheumatology Division Department of Rheumatology School of Medicine Hospital das Clínicas, University of São Paulo Brazil

## Abstract

Defective regulation of apoptosis may play a role in the development of autoimmune diseases. Fas and Bcl-2 proteins are involved in the control of apoptosis. The aims of this study were to determine the expression of Fas antigen and Bcl-2 protein on peripheral blood T and B lymphocytes from patients with juvenile-onset systemic lupus erythematosus (JSLE), juvenile rheumatoid arthritis (JRA) and juvenile dermatomyositis (JDM). Thirty-eight patients with JSLE, 19 patients with JRA, 10 patients with JDM and 25 healthy controls entered the study. Freshly isolated peripheral blood mononuclear cells (PBMC) were stained for lymphocyte markers CD3, CD4, CD8, CD19 and for Fas and Bcl-2 molecules. Expressions were measured by three-color flow cytometry. Statistical analysis was performed using Kruskal–Wallis test. Percentages of freshly isolated T lymphocytes positively stained for Fas protein from JSLE patients were significantly increased compared to healthy controls, patients with JRA and patients with JDM. Percentages of B lymphocytes positive for Fas from JSLE patients were higher than healthy controls and JRA patients. In addition, Fas expression on T cells from patients with JRA was increased compared to JDM patients. Otherwise, Fas expression on T and B cells from JRA and JDM patients were similar to healthy controls. MFI of Bcl-2 positive T lymphocytes from JSLE patients were significantly increased compared to healthy controls and JRA patients. MFI of Bcl-2 protein on B lymphocytes from JSLE patients was similar to healthy controls and patients with JRA and JDM. Bcl-2 expression did not differ between JRA and JDM patients and healthy controls. In conclusion, increased expression of Fas and Bcl-2 proteins observed in circulating T and B lymphocytes from patients with JSLE, but not from patients with JRA and JDM, suggests that abnormalities of apoptosis may be related to the pathogenesis of JSLE and probably are not a result of chronic inflammation.


*Clinical & Developmental Immunology*, June–December 2006; 13(2–4): 283–287

Taylor & Francis

Taylor & Francis Group

### Increased Fas and Bcl-2 expression on peripheral blood T and B lymphocytes from juvenile-onset systemic lupus erythematosus, but not from juvenile rheumatoid arthritis and juvenile dermatomyositis†

BERNADETE L. LIPHAUS[1], MARIA H.B. KISS[1], SOLANGE CARRASCO[2], & CLAUDIA GOLDENSTEIN-SCHAINBERG[2]

[1]*Rheumatology Unit, Department of Pediatrics, School of Medicine, Children's Institute, University of São Paulo, Brazil, and* [2]*Rheumatology Division, Department of Rheumatology, School of Medicine, Hospital das Clínicas, University of São Paulo, Brazil*

**Abstract**

Defective regulation of apoptosis may play a role in the development of autoimmune diseases. Fas and Bcl-2 proteins are involved in the control of apoptosis. The aims of this study were to determine the expression of Fas antigen and Bcl-2 protein on peripheral blood T and B lymphocytes from patients with juvenile-onset systemic lupus erythematosus (JSLE), juvenile rheumatoid arthritis (JRA) and juvenile dermatomyositis (JDM). Thirty-eight patients with JSLE, 19 patients with JRA, 10 patients with JDM and 25 healthy controls entered the study. Freshly isolated peripheral blood mononuclear cells (PBMC) were stained for lymphocyte markers CD3, CD4, CD8, CD19 and for Fas and Bcl-2 molecules. Expressions were measured by three-color flow cytometry. Statistical analysis was performed using Kruskal–Wallis test. Percentages of freshly isolated T lymphocytes positively stained for Fas protein from JSLE patients were significantly increased compared to healthy controls, patients with JRA and patients with JDM. Percentages of B lymphocytes positive for Fas from JSLE patients were higher than healthy controls and JRA patients. In addition, Fas expression on T cells from patients with JRA was increased compared to JDM patients. Otherwise, Fas expression on T and B cells from JRA and JDM patients were similar to healthy controls. MFI of Bcl-2 positive T lymphocytes from JSLE patients were significantly increased compared to healthy controls and JRA patients. MFI of Bcl-2 protein on B lymphocytes from JSLE patients was similar to healthy controls and patients with JRA and JDM. Bcl-2 expression did not differ between JRA and JDM patients and healthy controls. In conclusion, increased expression of Fas and Bcl-2 proteins observed in circulating T and B lymphocytes from patients with JSLE, but not from patients with JRA and JDM, suggests that abnormalities of apoptosis may be related to the pathogenesis of JSLE and probably are not a result of chronic inflammation.

**Keywords:** *Juvenile systemic lupus erythematosus, juvenile rheumatoid arthritis, juvenile dermatomyositis, Fas antigen, Bcl-2 protein, apoptosis*

### Introduction

Juvenile systemic lupus erythematosus (JSLE), juvenile rheumatoid arthritis (JRA) and juvenile dermatomyositis (JDM) are autoimmune diseases with high morbidity and mortality in childhood (Behrens et al. 1997; Andrade et al. 2000; Szodoray et al. 2003). These autoimmune diseases present dysfunction of T and B cells causing tissue infiltration of mononuclear cells and macrophages, tissue damage and production of autoantibodies (Behrens et al. 1997; Andrade et al. 2000; Szodoray et al. 2003). Their etiopathogenesis are not yet understood and it is possible that abnormalities of apoptosis at the stage of lymphocytes differentiation may be related to the presence of autoreactive T and B cells on peripheral blood (Behrens et al. 1997; Andrade et al. 2000; Kamradt and Mitchinson 2001; Szodoray et al. 2003). Additionally, increased rates of apoptotic cells may lead to the presence of great amounts of nuclear antigens at the extracellular tissue. These autoantigens can thus be presented to auto-reactive lymphocytes and drive to an autoimmune disease (Casciola-Rosen et al. 1994; Emlen et al. 1994; Wyllie 1997; Andrade et al. 2000; Smolewska et al. 2003).

Correspondence: B. L. Liphaus, Rheumatology Unit, School of Medicine, Children's Institute, Rua Alves Guimarães, 642/60, Jardim América, São Paulo, São Paulo, Brazil, CEP: 05410-001. Tel: 55 11 3898 1078. Fax: 55 11 3898 1078. E-mail: bernadll@icr.hcnet.usp.br

†Supported by a grant from FAPESP 03/00803-4.

ISSN 1740-2522 print/ISSN 1740-2530 online © 2006 Taylor & Francis
DOI: 10.1080/17402520600877786

Fas (APO1/CD95) is an apoptosis-promoting cell surface receptor. Mutations of *Fas* gene in MRL/Ipr mice lead to the development of a lymphoproliferative syndrome including features resembling lupus (Rieax-Laucat et al. 1995; Drappa et al. 1996). Increased Fas expression has been reported on peripheral T and B cells from patients with lupus, on synovial fluid cells from patients with rheumatoid arthritis and on muscle fibres from patients with dermatomyositis (Ohsako et al. 1994; Amasaki et al. 1995; Behrens et al. 1997; Sioud et al. 1998; Sugiura et al. 1999; Bijl et al. 2001). Bcl-2 protein inhibits apoptosis (Ivanovska et al. 2004). There is now considerable evidence that Bcl-2 expression is enhanced in peripheral T cells from patients with lupus (Strasser et al. 1991; Aringer et al. 1994; Rose et al. 1994; Mehrian et al. 1998; Falcini et al. 1999). Szodoray et al. (2003) observed increased expression of Bcl-2 on peripheral lymphocytes from patients with rheumatoid arthritis. In addition, increased expression of Bcl-2 was reported on muscle fibres from patients with dermatomyositis (Behrens et al. 1997; Falcini et al. 2000). Furthermore, the relation between inducers and inhibitors of apoptosis may differ according to cell type, to autoimmune disease and to age of disease onset.

In patients with juvenile systemic lupus erythematosus, JRA and JDM few number of studies concerning apoptosis and lymphocyte expression of Fas and Bcl-2 proteins have been performed. The aims of the present study were to examine the expression of Fas and Bcl-2 proteins in freshly isolated T and B lymphocytes from patients with JSLE, JRA and JDM.

### Patients and methods

Thirty-eight consecutive JSLE patients (32 females and 6 males; ages 8.2–20.9 years, mean age 14.0 years; mean disease duration 3.2 years) fulfilling at least four of the ACR revised criteria for lupus (Tan et al. 1982; Hochberg 1997), 19 patients with JRA (11 females and 8 males; ages 4.3–18.4 years, mean age 10.8 years) meeting the ACR criteria (Brewer et al. 1977) and 10 patients with JDM (4 females and 6 males; ages 3.4–13.2 years, mean age 9.3 years) meeting the criteria established by Bohan and Peter (1975) entered the study. Patients were recruited from the Rheumatology Unit, Children's Institute, School of Medicine, University of São Paulo, from April 2004 through March 2005. At disease onset all patients were younger than 16 years. Twenty-five sex- and age-matched healthy controls were also included in the study. Informed consent was obtained from all parents and the study was approved by the local ethics committee.

JSLE patients were basically treated with oral prednisone (0.5–1 mg/Kg/day) and 16 patients with severe disease were using immunosuppressive medication. Three JRA patients were without treatment, 15 were receiving nonsteroidal anti-inflammatory drugs and 10 were using oral prednisone associated with methotrexate or cyclosporine. Four JDM patients were without treatment, 5 were using oral prednisone associated with methotrexate or cyclosporine and one was using only methotrexate.

### *Immunofluorescence staining*

Immediately after venous blood was collected in EDTA tubes, peripheral blood mononuclear cells (PBMC) were separated by total blood lysis (Marti et al. 2001). Expression of Fas antigen and Bcl-2 protein on T and B lymphocytes was evaluated by three- and two-color immunofluorescence analysis.

Surface and cytoplasmatic staining were performed with the following monoclonal antibodies: Fluorescein isothiocyanate (FITC) conjugated monoclonal antibodies against Fas (Becton-Dickinson-Pharmigen (BD) mouse IgG1—555673), FITC-labeled anti-Bcl-2 (BD hamster IgG2a—554234), phycoerythrine (PE) conjugated monoclonal antibodies against CD4 (BD mouse IgG1—555347), PE-labeled anti-CD8 (BD mouse IgG1—555635), PE-labeled anti-CD19 (BD mouse IgG1—555413) and anti-CD3-Cy5 (BD mouse IgG1—555334).

After one wash with phosphate buffered saline (PBS) supplemented with 0.1% sodium azide, $1 \times 10^6$/ml cells were resuspended in 50 μl of PBS containing 2% fetal bovine serum (FBS) and stained with CD4, CD8, CD19, CD3 and Fas monoclonal antibodies. After 20-minute incubation in the dark, cells were lysed with 15-minute incubation in 10% FACS lysing solution and washed two times with PBS and resuspended using 1% paraformaldehyde prior to flow cytometric analysis.

For Bcl-2 protein intracytoplasmatic staining, cell membranes were permebilized by incubation with 4% paraformaldehyde and 10% FACS lysing solution for 10 min, washed twice with 0.5% PBS-Tween and FITC-labeled Bcl-2 was added. The CD4, CD8, CD19 and CD3 staining and other procedures were similar to Fas staining. Isotype controls were included in all experiments.

### *Flow cytometric analysis*

Immediately after staining procedure, flow cytometry analysis was done on a BD FAScan. Accurate physical and immunological gating was performed using CD3-Cy5. Positive and negative controls were run for each sample in order to standardize FACS procedures. During the 11-month duration of this study there was no realignment of the cytometer. Instrument settings were kept constant and fluorescence alignment constancy for forward scatter, green fluorescence and red fluorescence were

Table I. Percentage of lymphocytes positive stained for Fas—mean ± SD.

| Subjects | N° | CD3 + T cells | CD4 + T cells | CD8 + T cells | CD19 + B cells |
|---|---|---|---|---|---|
| JSLE | 38 | 43.7 ± 10.3 | 20.3 ± 6.7 | 21.5 ± 9.6 | 2.1 ± 1.4 |
| JRA | 19 | 34.9 ± 10.6* | 18.2 ± 3.9 | 17.3 ± 9.7* | 1.4 ± 0.7* |
| JDM | 10 | 24.9 ± 13.4* | 13.0 ± 4.0* | 12.7 ± 13.5* | 1.6 ± 0.8 |
| Healthy controls | 25 | 31.2 ± 9.1* | 17.9 ± 6.3 | 12.5 ± 5.9* | 1.2 ± 0.5* |

JSLE, juvenile systemic lupus erythematosus; JRA, juvenile rheumatoid arthritis; JDM, juvenile dermatomyositis; *$p < 0.05$ JSLE vs. healthy controls, JRA or JDM; Kruskal–Wallis test.

monitored before each analysis. Forward and site scatter profiles were identical for all patients and controls analysed in parallel in each session. Ten thousand lymphocytes were analysed per sample and double positive cells identified by dot-plot histograms. The results were expressed as percentage of double staining cells in relation to the cellular total and as mean fluorescence intensity (MFI).

### *Statistical analysis*

Data were analysed using SPSS for windows. To determine if there were significant differences between lymphocyte Fas and Bcl-2 expressions from patients and healthy controls the nonparametric Kruskal–Wallis test was used (Rosner 2000). *P* values less than 0.05 were considered statistically significant.

### Results

### *Expression of Fas antigen*

Percentages of freshly isolated lymphocytes positively stained for Fas antigen from JSLE patients were significantly increased compared to healthy controls on CD3 +, on CD8 + T cells and on CD19 + B cells. Fas expression on lymphocytes from JSLE patients were also increased compared to JRA patients on CD3 +, CD8 + T cells and on CD19 + B and compared to JDM patients on CD3 +, CD4 + and CD8 + T cells (Table I). JRA patients presented percentages of lymphocytes positively stained for Fas antigen significantly increased compared to JDM patients on CD3 + (34.9 ± 10.6 vs. 24.9 ± 13.4; $p = 0.02$), on CD4 + (18.2 ± 3.9 vs. 13.0 ± 4.0; $p = 0.005$) and on CD8 + T cells (17.3 ± 9.7 vs. 12.7 ± 13.5; $p = 0.03$). Otherwise, percentages of positive cells and MFI of Fas antigen from patients with JRA and JDM were similar to healthy controls (Table I).

### *Expression of Bcl-2 protein*

The density of Bcl-2 protein (MFI) on lymphocytes from JSLE patients were significantly increased ($p < 0.05$) compared to healthy controls on CD3 +, on CD4 + and on CD8 + T cells, but not on CD19 + B cells. MFI of Bcl-2 was also increased on lymphocytes from JSLE patients compared to JRA patients on CD3 +, CD4 + and CD8 + T cells, but there was no difference compared to JDM patients (Table II). Bcl-2 protein expression on T and B lymphocytes from patients with JRA and JDM did not differ from healthy controls. And there was no difference between Bcl-2 expression on T and B cells from JRA and JDM patients (Table II).

### Discussion

The present study showed increased expression of Fas and Bcl-2 proteins on peripheral T and B cells from patients with JSLE, but not from patients with JRA and JDM.

Fas and Bcl-2 proteins play a significant role in lymphocyte proliferation, survival and apoptosis (Kamradt and Mitchinson 2001). It is not clear how apoptosis relates to the pathogenesis of autoimmune diseases (Andrade et al. 2000). However, there is a hypothesis that accelerated apoptosis of circulating lymphocytes might lead to the release of increased amounts of intact nuclear antigens that can drive to an autoimmune response and to combine with autoantibodies to form immune complexes (Wyllie 1997; Andrade et al. 2000). Some studies have shown that lymphocytes

Table II. MFI of Bcl-2 positive lymphocytes—mean ± SD.

| Subjects | N° | CD3 + T cells | CD4 + T cells | CD8 + T cells | CD19 + B cells |
|---|---|---|---|---|---|
| JSLE | 38 | 28.8 ± 8.4 | 28.6 ± 8.2 | 29.4 ± 9.3 | 25.5 ± 9.6 |
| JRA | 19 | 24.4 ± 4.4* | 23.6 ± 4.6* | 23.9 ± 5.7* | 22.3 ± 5.3 |
| JDM | 10 | 26.9 ± 8.4 | 25.7 ± 7.5 | 29.5 ± 8.9 | 22.3 ± 4.8 |
| Healthy controls | 25 | 26.0 ± 7.2* | 25.9 ± 7.2* | 25.5 ± 6.7* | 23.6 ± 5.6 |

JSLE, juvenile systemic lupus erythematosus; JRA, juvenile rheumatoid arthritis; JDM, juvenile dermatomyositis; *$p < 0.05$ JSLE vs. healthy controls, JRA or JDM; Kruskal–Wallis test.

from patients with lupus and JRA undergo apoptosis at significant greater rates than lymphocytes from healthy controls (Emlen et al. 1994; Chan et al. 1997; Smolewska et al. 2003).

Experimental studies revealed that MRL/Ipr mice have a mutation of *Fas* gene that prevents expression and cell death. These mice develop a lymphoproliferative syndrome including features resembling human lupus (Rieax-Laucat et al. 1995; Drappa et al. 1996). Transgenic mice overexpressing Bcl-2 protects B cells against cell death and it promotes the development of a lupus-like autoimmune syndrome with nephritis and autoantibodies (Strasser et al. 1991; Rose et al. 1994). It is likely that abnormal expression of Fas antigen can increase the exposure of hidden antigens and abnormal expression of Bcl-2 inhibits removal of auto-reactive cells, thus leading to an autoimmune disease (Andrade et al. 2000). Although recent data suggest a relation between increased Fas and Bcl-2 expression and lupus pathogenesis, it is not know if these abnormalities are primary or due to chronic inflammation and whether they can occur in other autoimmune diseases.

The present study demonstrated increased percentages of T and B lymphocytes positively stained for Fas antigen from JSLE patients compared to healthy controls, JRA and JDM patients. Increased expression of Fas antigen on CD3 + T lymphocytes was also showed by Courtney et al. (1999). Unlike the present results, Amasaki et al. (1995) observed increased expression of Fas antigen on CD4 + and CD8 + T cells from lupus patients compared to healthy controls. Bijl et al. (2001) also showed a higher expression of Fas protein on B lymphocytes from patients with lupus. And Ohsako et al. (1994) observed increased Fas expression on B cells from lupus patients with active disease. Szodoray et al. (2003) observed an underexpression of pro-apoptotic proteins Fas and Bax on lymphocytes from patients with rheumatoid arthritis. Sioud et al. (1998) demonstrated increased expression of Fas protein on synovial fluid CD3 + T cells from patients with rheumatoid arthritis and JRA. On dermatomyositis patients, Behrens et al. (1997) and Sugiura et al. (1999) observed increased expression of Fas in muscle fibres and inflammatory cells. In addition, we observed increased expression of Fas protein on CD3 +, on CD4 + and on CD8 + T cells from patients with JRA compared to JDM patients. Fas antigen is the main protein related to apoptosis induction. Apoptosis might usually be induced in activated lymphocytes, which would lead to a more rapid turnover of these cells and a greater exposure of autoantigens (Kamradt and Mitchinson 2001). This study is also in accordance to the suggestion that increased expression of Fas antigen and enhancement of apoptotic material may be a hallmark of lupus and probably not related to chronic inflammation (Ohsako et al. 1994; Amasaki et al. 1995; Bijl et al. 2001).

Expression of apoptosis-inhibitory protein Bcl-2 on freshly isolated lymphocytes has been a matter of controversy. Some investigators have shown increased Bcl-2 expression in T cells, but not on B cells while others demonstrated unaltered Bcl-2 quantities in unfractionated lymphocytes in adult patients with lupus (Hockenbery et al. 1993; Aringer et al. 1994; Mehrian et al. 1998; Falcini et al. 1999; Ivanovska et al. 2004). The present study showed a significantly increased Bcl-2 expression on all T lymphocytes subsets compared to healthy controls. These results are similar to that observed by Aringer et al. (1994) and Ohsako et al. (1994). Otherwise, Rose et al. (1995) observed a similar expression of Bcl-2 protein on lupus patients and healthy controls and Chan et al. (1997) demonstrated decreased expression of Bcl-2 protein on lymphocytes from patients with lupus. On juvenile-onset SLE patients Falcini et al. (1999) also observed a higher expression of Bcl-2 protein on T lymphocytes, but not on B cells. Szodoray et al. (2003) observed increased expression of Bcl-2 protein on lymphocytes from patients with rheumatoid arthritis, possibly resulting in increased survival of auto-reactive lymphocytes. Behrens et al. (1997) observed increased expression of Bcl-2 in muscle fibres and inflammatory cells form patients with dermatomyositis. Indeed, this study supports a possible role of increased expression of Bcl-2 on JSLE pathogenesis considering that it may lead to longevity and persistence of auto-reactive lymphocyte clones (Hockenbery et al. 1993; Andrade et al. 2000; Kamradt and Mitchinson 2001).

Interestingly, Fas and Bcl-2 expressions on T and B lymphocytes from JRA and JDM patients were indistinguishable from their expression on lymphocytes from healthy controls, suggesting that the altered Fas and Bcl-2 expression observed on lymphocytes may be at least somewhat specific for JSLE. However, whether the abnormalities of apoptosis observed herein in JSLE patients could be considered a primary pathogenic event or, alternatively, a secondary epiphenomenon it remains to be elucidated. Comparing our results to others studies, it can be suggest that on JRA and JDM patients the expression of apoptotic regulated proteins is important in determining local inflammation such as chronic synovitis and muscle lesion, but it is not a systemic alteration observed on peripheral lymphocytes like on patients with JSLE (Behrens et al. 1997; Sioud et al. 1998; Sugiura et al. 1999; Falcini et al. 2000).

In conclusion, the results presented herein showed that a dysregulation of apoptosis occurs in JSLE patients somewhat similarly to adult patients with lupus, considering the increased expression of Fas and Bcl-2 proteins on T and B lymphocytes. These data also suggest that these alterations are somewhat exclusive to JSLE patients and not only related to chronic inflammation.

### References

Amasaki Y, Kobayashi S, Takeda T, Ogura N, Jodo S, Nakabayashi T, Tsutsumi A, Fujisaku A, Koike T. 1995. Up-regulated expression of Fas antigen (CD95) by peripheral naïve and memory T cell subsets in patients with systemic lupus erythematosus (SLE): A possible mechanism for lymphopenia. Clin Exp Immunol 22(2):245–250.

Andrade F, Casciola-Rosen L, Rosen A. 2000. Apoptosis in systemic lupus erythematosus. Clinical implications. Rheum Dis Clin of North Am 26(2):215–227.

Aringer M, Wintersberger W, Steiner CW, Kiener H, Presterl E, Jaeger U, Smolen JS, Graninger WB. 1994. High levels of Bcl-2 protein in circulating T lymphocytes, but not B lymphocytes of patients with systemic lupus erythematosus. Arthritis Rheum 10:1423–1430.

Behrens L, Bender A, Johnson MA, Hohlfed R. 1997. Cytotoxic mechanisms in inflammatory myopaties. Co-expression of Fas and protective Bcl-2 in muscle fibres and inflammatory cells. Brain 120(Pt6):929–938.

Bijl M, Horst G, Limburg PC, Kallenberg CG. 2001. Fas expression on peripheral blood lymphocytes in systemic lupus erythematosus (SLE): Relation to lymphocyte activation and disease activity. Lupus 10(12):866–872.

Bohan A, Peter JB. 1975. Polymiositis and dermatomyositis (parts 1 and 2). N Engl J Med 292:344–347–403–407.

Brewer EJ, Bass JC, Baum J, et al. 1977. Current proposed revision of JRA criteria. Arthritis Rheum 20:195.

Casciola-Rosen LA, Anhalt G, Rosen A. 1994. Autoantigens targeted in systemic lupus erythematosus are clustered in two populations of surface structures on apoptotic keratinocytes. J Exp Med 179:1317–1330.

Chan EY, Ko SC, Lau CS. 1997. Increased rate of apoptosis and decreased expression of Bcl-2 protein in peripheral blood lymphocytes from patients with active systemic lupus erythematosus. Asian Pac J Allergy Immunol 15(1):3–7.

Courtney PA, Crockard AD, Williamson K, Mcconnell J, Kennedy RJ, Bell AL. 1999. Lymphocyte apoptosis in systemic lupus erythematosus: Relationships with Fas expression, serum soluble Fas and disease activity. Lupus 8:508–513.

Drappa J, Vaishnaw AK, Sullivan KE, Chu JL, Elkon KB. 1996. *Fas* gene mutations in the Canele-Smith syndrome, an inherited lymphoproliferative disorder associated with autoimmunity. N Engl J Med 335:1643–1649.

Emlen W, Niebur J, Kadera R. 1994. Accelerated *in vitro* apoptosis of lymphocytes from patients with systemic lupus erythematosus. J Immunol 152:3685–3692.

Falcini F, Azzari C, Gelli VAMG, Luchetti M, Gabrielli A, Calzolari A, Pignone A, Generini S, Matucci Cerinic C. 1999. Reduction of Bcl-2 in T cells during immunosuppressive therapy in patients with severe juvenile onset systemic lupus erythematosus. Clin Immunol 93(1):59–64.

Falcini F, Calzolari A, Generini S, Pignone A, Simonini G, Zulian F, Matucci-Cerinic M. 2000. Bcl-2, p53 and c-myc expression in juvenile dermatomyositis. Clin Exp Rheumtol 18(5):643–646.

Hochberg MC. 1997. Updating the American College of Rheumatology resived criteria for the classification of systemic lupus erythematosus. Arthritis Rheum 40:1725.

Hockenbery DM, Oltvai ZN, Yin XM, Milliman CL, Korsmeyer SJ. 1993. Bcl-2 functions in an antioxidant pathway to prevent apoptosis. Cell 75:241–251.

Ivanovska I, Galonek HL, Hildeman DA, Hardwick JM. 2004. Regulation of cell death in the lymphoid system by Bcl-2 family proteins. Acta Haematol 111:42–55.

Kamradt T, Mitchinson NA. 2001. Advances in immunology: Tolerance and autoimmunity. N Engl J Med 344(9):655–664.

Marti GE, Steler-Stevenson M, Bleesing JJH, Fleisher TA. 2001. Introduction to flow cytometry. Sem Hematol 38(2):93–99.

Mehrian R, Quismorio FP, Jr, Strassmann G, et al. 1998. Synergistic effect between IL-10 and Bcl-2 genotypes in determining susceptibility to systemic lupus erythematosus. Arthritis Rheum 41(4):596.

Ohsako S, Hara M, Harigai M, Fukasawa C, Kashiwagaki S. 1994. Expression and function of Fas antigen and Bcl-2 in human systemic lupus erythematosus lymphocytes. Clin Immunol Immunophatol 73(1):109–114.

Rieax-Laucat F, Le Deist F, Hivroz C, Roberts IAG, Debatin KM, Fischer A, De Villartay JP. 1995. Mutations in Fas associated with human lymphoproliferative syndrome and autoimmunity. Science 268:1347–1349.

Rose LM, Latchman DS, Isenberg DA. 1994. Bcl-2 and Fas, molecules which influence apoptosis. A possible role in systemic lupus erythematosus? Autoimmunity 17(4):271–278.

Rose LM, Latchman DS, Iseberg DA. 1995. Bcl-2 expression is unaltered in unfractionated peripheral blood mononuclear cells in patients with systemic lupus erythematosus. Br J Rheumatol 34(4):316–320.

Rosner B. 2000. Fundamentals of biostatistics. 5th ed. Duxburg, CA: Thomsom Learning.

Sioud M, Mellbye O, Forre O. 1998. Analysis of the NF-kappa B p65 subunit, Fas antigen, Fas ligand and Bcl-2 related proteins in the synovium of RA and polyarticular JRA. Clin Exp Rheumatol 16(2):125–134.

Smolewska E, Brozik H, Smolewski P, Biernacka-Zielinska M, Darzynkiewicz Z, Stanczyk J. 2003. Apoptosis of peripheral blood lymphocytes in patients with juvenile idiopathic arthritis. Ann Rheum Dis 62(8):761–763.

Strasser A, Whittingham S, Vaux DL, et al. 1991. Enforced Bcl-2 expression in B lymphoid cells prolongs antibody responses and elicits autoimmune disease. Proc Natl Acad Sci USA 88:8661–8665.

Sugiura T, Murakawa Y, Nagai A, Kondo M, Kobayashi S. 1999. Fas and Fas ligand interaction induces apoptosis in inflammatory myopathies: CD4 + T cells cause muscle cell injury directly in polymyositis. Arthritis Rheum 42(2):291–298.

Szodoray P, Jellestad S, Nakken B, Brun JG, Jonsson R. 2003. Programmed cell death in rheumatoid arthritis peripheral blood T-cell subpopulations determined by laser scanning cytometry. Lab Invest 83(12):1839–1848.

Tan EM, Cohen AS, Frils JF, Masi AT, Mcshane D, Rothifield NF, Scholler J, Tatal N, Winchester R. 1982. The 1982 revised criteria for the classification of SLE. Arthritis Rheum 25:1271–1277.

Wyllie AH. 1997. Apoptosis: An overview. Br Med Bull 53:451–465.

